# Supplementary material for: Sacubitril/Valsartan Combination Partially Improves Cardiac Systolic, but Not Diastolic, Function through β-AR Responsiveness in a Rat Model of Type 2 Diabetes
Source: Int J Mol Sci. 2024 Oct 2;25(19):10617. doi: 10.3390/ijms251910617 (PMC11476658; doi:10.3390/ijms251910617)
Supplement: Supplementary file 1 [file ijms-25-10617-s001.zip › ijms-3127195-supplementary.pdf]

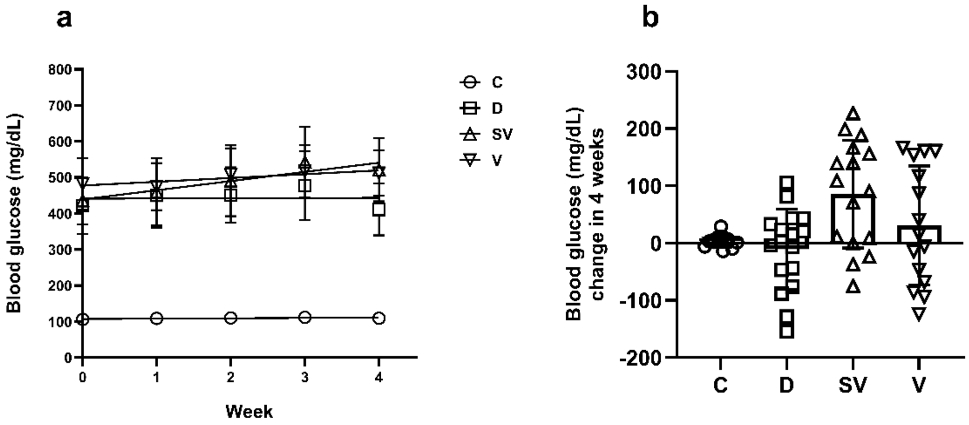

Supplementary Figure S1. Blood glucose levels during treatment. **(a)** Weekly measured blood glucose levels starting after treatment (means ± SD). **(b)** Blood glucose change. C, Control (n=13); D, Diabetic (n=17); SV, Sacubitril/valsartan treated diabetic (n=16); V, Valsartan treated diabetic (n=15); each data point is one animal.

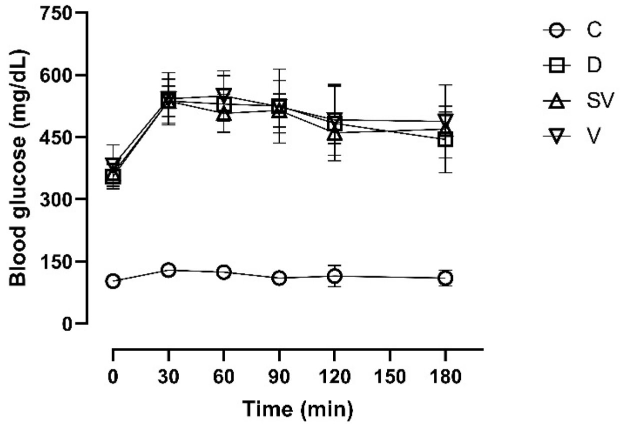

Supplementary Figure S2. Time course blood glucose levels during OGTT. C, Control (n=8); D, Diabetic (n=7); SV, Sacubitril/valsartan treated diabetic (n=8); V, Valsartan treated diabetic (n=9); values show as means ± SD.

Supplementary Table S1. Blood glucose levels at each time point during OGTT
C, Control (n=8); D, Diabetic (n=7); SV, Sacubitril/valsartan treated diabetic (n=8); V, V, Valsartan treated diabetic (n=9). ****, p<0.0001 compared to control. Values shown as means ± SD.

	C (n=8)	D (n=7)	SV (n=8)	V (n=9)
**0. min BG (mg/dL)**	102.38 ± 7.95	354.71 ± 29.25****	362.13 ± 24.19	381.33 ± 50.14
**30. min BG (mg/dL)**	128.88 ± 10.36	536.86 ± 53.06****	536.13 ± 36.72	542.00 ± 63.31
**60. min BG (mg/dL)**	124.13 ± 13.16	529.14 ± 68.54****	507.25 ± 45.60	549.11 ± 60.58
**90. min BG (mg/dL)**	110.13 ± 12.81	524.86 ± 89.11****	513.88 ± 40.02	523.00 ± 63.04
**120. min BG (mg/dL)**	114.63 ± 25.49	482.57 ± 90.48****	460.13 ± 25.96	491.22 ± 85.40
**180. min BG (mg/dL)**	109.88 ± 18.94	444.00 ± 79.86****	469.13 ± 41.35	487.33 ± 88.40

Supplementary Table S2. Fasting blood glucose and insulin levels prior to OGTT and HOMA-IR values
C, Control (n=5); D, Diabetic (n=5); SV, Sacubitril/valsartan treated diabetic (n=6); V, Valsartan treated diabetic (n=5). ****, p<0.0001 compared to control. Values shown as means ± SD,

	C (n=5)	D (n=5)	SV (n=6)	V (n=5)
**Fasting blood glucose (mg/dL)**	102.00 ± 8.34	350.40 ± 27.49****	365.00 ± 24.39	400.20 ± 62.54
**Fasting insulin (mIU/L)**	10.21 ± 0.77	10.77 ± 0.96	10.30 ± 0.86	10.36 ± 0.63
**HOMA-IR**	2.56 ± 0.20	9.27 ± 0.45****	9.29 ± 1.18	10.22 ± 1.74

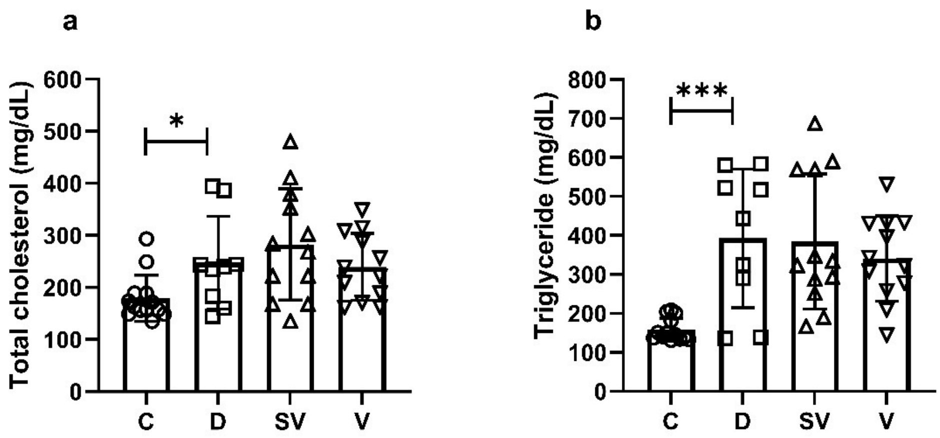

Supplementary Figure S3. Plasma lipid levels. **(a)** Total cholesterol levels. **(b)** Triglyceride levels. C, Control (n=13); D, Diabetic (n=9); SV, Sacubitril/valsartan treated diabetic (n=12); V, Valsartan treated diabetic (n=12). *, p<0.05; ***, p<0.001 compared to control. Each data point represents one animal.

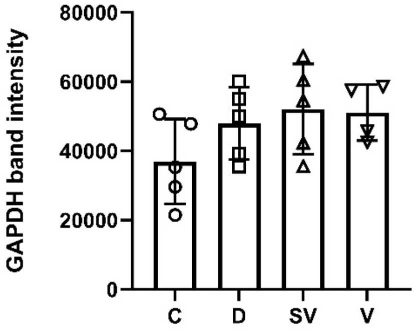

Supplementary Figure S4. GAPDH validation as a housekeeping gene. C, Control (n=5); D, Diabetic (n=5); SV, Sacubitril/valsartan treated diabetic (n=5); V; Valsartan treated diabetic (n=4). Each datapoint represents one animal.
